# Overexpression of *Loose Plant Architecture 1* increases planting density and resistance to sheath blight disease via activation of *PIN‐FORMED 1a* in rice

**DOI:** 10.1111/pbi.13072

**Published:** 2019-02-19

**Authors:** Qian Sun, Tian Ya Li, Dan Dan Li, Zi Yuan Wang, Shuang Li, Dao Pin Li, Xiao Han, Jing Miao Liu, Yuan Hu Xuan

**Affiliations:** ^1^ College of Plant Protection Shenyang Agricultural University Shenyang China; ^2^ College of Life Science Yan'an University Yan'an Shaanxi China; ^3^ Department of Agricultural and Biological Technology WenZhou Agricultural Science Research Institute (WenZhou Vocational College of Science &Technology) Wenzhou China; ^4^ College of Biological Science and Engineering Fuzhou University Fuzhou China

**Keywords:** LPA1, tiller angle, sheath blight disease, PIN1a, rice

**Figure 1:**
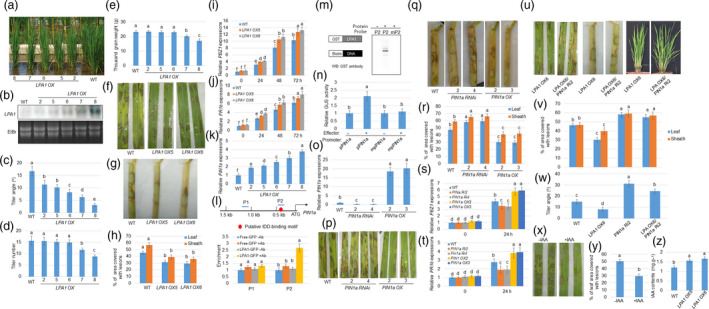
*LPA1* triggers *PIN1a* to regulate tiller angle and resistance to sheath blight disease (SBD). (a) 2‐month‐old wild‐type (WT) and *LPA1* overexpressors (OX; 2, 5, 6, 7 and 8) were aligned according to the degree of tiller angles. (b) *LPA1* expression levels in WT and LPA1 overexpressors were analysed by northern blot analysis. EtBr staining of rRNA was used as a loading control. Tiller angles (c) and number (d) from the lines shown in (a) are shown. Data indicate average ± standard deviation (SD) (*n* > 10). (e) Thousand grain weight from the WT and *LPA1 OX* lines were measured. Data indicate average ±SD (*n* = 6). Leaves (f) and sheath (g) from the WT and *LPA1 OX* lines (OX5 and OX6) were inoculated with *Rhizoctonia solani* AG1‐1A and were photographed after infection. Six leaves from each line were examined. Each experiment was performed in triplicate. (h) The lesion areas on the leaf or sheath surfaces of *R*. *solani* AG1‐1A‐infected tissues were examined. Data indicate average ±standard error (SE) (*n* > 10). *PBZ1* (i) and *PR1b* (j) expression levels in the WT and *LPA1 OX* lines (5 and 6) after 0, 24, 48 and 72 hours of *R. solani* AG1‐1A inoculation using Quantitative Reverse Transcription Polymerase Chain Reaction (qRT‐PCR). The experiments were performed in triplicate. (k) The expression levels of *PIN1a* were monitored in the WT and *LPA1 OX* lines (2, 5, 6, 7 and 8) using qRT‐PCR. The experiments were performed in triplicate. (l) Schematic diagram indicating location of the putative IDD‐binding motif (red circle) within 1.5 kb of *PIN1a* promoter and probes (P) used for chromatin immunoprecipitation (ChIP) assays. Relative ratios of immunoprecipitated DNA to input DNA were determined by qPCR. Input DNA was used to normalize the data. −Ab or +Ab: green fluorescent protein (GFP) antibody. Error bars represent ±SE (*n* = 3). (m) An electrophoretic mobility‐shift assay (EMSA) was conducted to evaluate *LPA1* affinities to P2 and mutated probe mP2. The probe was labelled with biotin and the band shifting was detected via western blot analysis using anti‐glutathione‐S‐transferase (GST) antibody. (n) A transient expression assay was conducted by co‐transfection with p35S: *LPA1* and each of the vectors expressing the beta‐glucuronidase gene (GUS) under the control of native (*pPIN1a*) and IDD‐binding motif‐mutated (*mpPIN1a*) *PIN1a* promoters in protoplast cells. The luciferase gene driven by the 35S promoter was used as an internal control to normalize GUS expression. Error bars represent ± SE (*n* = 6). (o) PIN1a expression level in WT, *PIN1a RNAi* lines (2 and 4) and *PIN1a OX* lines (2 and 3) was examined using qRT‐PCR. The experiments were performed in triplicate. Leaves (p) and sheath (q) from the WT, *PIN1a RNAi* lines (2 and 4) and *PIN1a OX* lines (2 and 3) were inoculated with *R*. *solani* AG1‐1A and were photographed after infection. The leaves and sheath from each line were examined, and the experiments were performed in triplicate. (r) The lesion areas on the leaf surfaces were examined for *R*. *solani* AG1‐1A‐infected leaves and sheath. Data indicate average ± SE (*n* > 10). *PBZ1* (s) and *PR1b* (t) expression levels in the WT, *PIN1a RNAi* lines (2 and 4) and *PIN1a OX* lines (2 and 3) after 0 and 48 hours of *R. solani* AG1‐1A inoculation using qRT‐PCR. The experiments were performed in triplicate. (u) *LPA1 OX6* and *LPA1 OX6/PIN1a RNAi2* (Ri2) double‐mutant leaves and sheath, respectively, were inoculated with *R*. *solani* AG1‐1A (left and middle) and 2‐month‐old *LPA1 OX6* and *LPA1 OX6/PIN1a Ri2* plants were photographed (right). (v) The lesion area on the leaf and sheath surface of WT, *LPA1 OX6*, *PIN1a Ri2* and *LPA1 OX6/PIN1a Ri2* plants was measured for *R*. *solani* AG1‐1A‐infected tissues. Data indicate average ± SE (*n* > 10). (w) Tiller angle of genetic combination between *LPA1 OX6* and *PIN1a Ri2* were analysed. More than 10 plants from segregated WT, *LPA1 OX6*, *PIN1a Ri2* and *LPA1 OX6/PIN1a Ri2* plants were used for measurement. Data indicate averages ±SE. (x) Leaves from 2‐month‐old WT plants with or without 100 nM IAA treatment for 3 days, were inoculated with *R*. *solani* AG1‐1A. (y) The lesion areas on the leaf surfaces of *R*. *solani* AG1‐1A‐infected leaves shown in (p) were examined. Data indicate averages ±SE (*n* > 10). (z) IAA content from the leaves of 2‐month‐old WT and *LPA1 OX* lines (OX5 and OX6) were measured. Vertical bars indicate average values ±SE (*n* = 3). One‐way analysis of variance (ANOVA) followed by Bonferroni's multiple comparison tests were performed to assess significant differences between more than two groups. Different letters above the bars denote statistically significant differences (*P* < 0.05).

Increasing yield and resistance to pathogens are important objectives in plant breeding. However, difficulties in breeding are encountered due to the antagonistic relationship between crop yield production and immunity pathways (Ning *et al*., 2017). Planting density and plant architecture are key factors determining crop yields in a given area. The plant traits of tiller and lamina joint angles have long attracted the attention of breeders due to their significant contributions to plant architecture by enhancing photosynthetic efficiency and facilitating enhanced planting density (Sakamoto *et al*., 2006; Wang and Li, 2008). Earlier reports have shown that Loose Plant Architecture 1 (*LPA1*), used for encoding an indeterminate domain (IDD) protein, negatively controls tiller and lamina joint angle in an expression level‐dependent manner (Liu *et al*., 2016; Wu *et al*., 2013). Overexpression of *LPA1* significantly decreased tiller angle and resulted in the development of erect leaves and the generation of severe lines at an angle of ~1/5 that of the tiller angle, relative to the wild‐type (WT) plants. Also, *LAP1* mRNA was highly accumulated in overexpressor lines and the levels were negatively associated with tiller angle (Figure 1a–c). Further inspection demonstrated that severe lines resulted in decreased tiller number and thousand grain weight, whereas the lines with moderate expressions sustained similar tiller numbers and thousand grain weight relative to the WT (Figure 1d,e), implying that moderate expression of *LPA1* increases planting density without impacting tiller number and seed weight.

To determine whether these lines not only increase planting density but also pathogen resistance, *Rhizoctonia solani* AG1‐1A, which is the cause of sheath blight disease (SBD), one of the major rice diseases, was inoculated to the leaves of the WT and *LPA1* overexpressors (OX5 and OX6, the tiller number and thousand grain weight of which were not impacted). SBD imperils rice throughout its growth cycle, from seedling to heading, and causes lesions on leaves, sheaths, and panicles that can decrease rice yield by 8%–50%, depending on disease severity (Savary *et al*., 1995, 2000). However, resistant cultivars and gene sources against SBD are currently lacking. Interestingly, *LPA1* overexpressors are less vulnerable to *R*. *solani* AG‐1 than WT plants (Figure 1f,g). 46% of the leaf area was covered with lesions in the WT, 30% in *LPA1 OX5,* and 29% in *LPA1 OX6*. While 54% of the sheath area was covered with lesions in the WT, 37.8% in *LPA1 OX5* and 38.6% in *LPA1 OX6* (Figure 1h), implying that *LPA1* overexpression enhanced plant resistance to SBD. Further examination indicated that expression of *PBZ1* and *PR1b*, two pathogen resistant genes were more highly induced in *LPA1 OXs* than in WT after inoculation of *R. solani* AG1‐1 (Figure 1i,j). Our earlier work demonstrated that hormonal signals play key roles in rice resistance to SBD (Yuan *et al*., 2018). In addition, *LPA1* positively controls the expressions of the auxin efflux carrier gene *PIN‐FORMED 1a* (*PIN1a*) in the lamina joint (Liu *et al*., 2016). Phenotypically, *PIN1a* knock‐down plants exhibited increased tiller angle whereas *PIN1a* overexpression lines slightly decreased tiller angle relative to that of the WT (Xu *et al*., 2005), similar to differences in comparisons between *LPA1* mutants and overexpressors for plant shape (Wu *et al*., 2013). Quantitative Reverse Transcription Polymerase Chain Reaction (qRT‐PCR) analysis identified that *LPA1* overexpression up‐regulated *PIN1a* expressions in leaves (Figure 1k). As *PIN1a* was positively regulated by *LPA1* and IDD proteins are known to function as a transcription factor (Kozaki *et al*., 2004), the *PIN1a* promoter sequences were examined to identify the presence of putative IDD‐binding motif. A single IDD‐binding motif was located within 1.5 kb of the *PIN1a* promoter (Figure 1l). To determine the binding affinity of *LPA1* to the IDD‐binding motif, a chromatin immunoprecipitation (ChIP) assay was conducted using 35S: green fluorescent protein (GFP) and 35S:*LPA1*:GFP transgenic plant calli. Without addition of GFP, the antibody was used as the control for the GFP antibody to immunoprecipitate DNA. Data showed that LPA1 bound to the P2, but not to the P1 region (Figure 1l). We further conducted an electrophoretic mobility shift assay (EMSA) to verify binding affinity of P2 to LPA1. We found that *LPA1* bound to biotin‐labelled P2; however, it failed to bind to the mutated probe mP2 that were detected by western blot analysis using GST antibody (Figure 1m). To verify whether these *cis*‐elements were responsible for the transcriptional initiation of *PIN1a* promoter by LPA1, we conducted transient expression assays using the protoplast system. Protoplast cells were co‐transformed with the 35S:*LPA1* plasmid and a vector expressing the beta‐glucuronidase gene (GUS) under the control of *pPIN1a* or *mpPIN1a*. In the mutated promoter (*mpPIN1a*), IDD‐binding motif sequences TTTGTCG were substituted by the sequence AAAAAAA. Using 35S:luciferase (LUC) as an internal control to normalize the transformation efficiency in each assay, protoplasts expressing *LPA1* had approximately twice the levels of activated *pPIN1a*; however, *LPA1* was unable to activate *mpPIN1a* (Figure 1m). These results show that LPA1 directly triggers *PIN1a* via promoter binding.

Since *PIN1a* is a target of *LPA1*, the role of *PIN1a* in resistance to SBD was examined. *PIN1a RNAi* lines and overexpression plants were used to evaluate the response of *PIN1a* to *R*. *solani* AG1‐1A. qRT‐PCR results showed that *PIN1a* level was obviously lower in *PIN1a RNAi* lines (Ri2 and Ri4) and higher in *PIN1a* overexpression lines (*OX2* and *OX3*) compared to wild‐type one (Figure 1o). Furthermore, the results demonstrated that *PIN1a RNAi* lines (Ri2 and Ri4) were more vulnerable, whereas overexpression lines (*OX2* and *OX3*) were less vulnerable to *R*. *solani* AG1‐1A (Figure 1p,q). 47% of the leaf area was covered with lesions in the WT, 58% in *PIN1a Ri2*, 59% in *PIN1a Ri4*, 30% in *PIN1a OX2,* and 29% in *PIN1a OX3*. While 58% of the sheath area was covered with lesions in the WT, 64% in *PIN1a Ri2*, 65% in *PIN1a Ri4*, 40% in *PIN1a OX2,* and 41% in *PIN1a OX3* (Figure 1r), implying that *PIN1a* positively controls rice resistance to SBD, similar to the degree of *LPA1* regulation on SBD resistance. In addition, *PBZ1* and *PR1b* expression levels were less induced in *PIN1a RNAi* lines while more highly induced in *PIN1a OXs* than in WT after inoculation of *R. solani* AG1‐1 (Figure 1s,t). Next, we investigated whether *LPA1* controls planting density and resistance to SBD via initiation of *PIN1a* by genetic combination between *LPA1 OX6* and *PIN1a Ri2*. Analysis of the inoculation of *R*. *solani* with AG1‐1A showed that *LPA1 OX6* is less vulnerable, whereas *PIN1a Ri2* is more vulnerable to SBD. Furthermore, *PIN1a Ri2* enhanced *LAP1 OX6* vulnerability to SBD, and *PIN1a Ri2* and *LPA1 OX6/PIN1a Ri2* exhibited a similar degree of vulnerability response to *R*. *solani* AG1‐1A. 47% of the leaf area was covered with lesions in the WT, 30% in *LPA1 OX6,* 58% in *PIN1a Ri2,* and 56% in *LPA1 OX6/PIN1a Ri2*. While 48% of the leaf area was covered with lesions in the WT, 40% in LPA1 *OX6,* 59% in *PIN1a Ri2,* and 57% in *LPA1 OX6/PIN1a R i2* (Figure 1u,v). In parallel, tiller angle was compared between the WT, *LPA1 OX6*,* PIN1a Ri2,* and *LPA1 OX6/PIN1a Ri2* from the same siblings. The *LPA1 OX6* decreased tiller angle whereas *PIN1a Ri2* enlarged tiller angle relative to that of the WT. *LPA1 OX6/PIN1a Ri2* exhibited enhanced tiller angle relative to *LPA1 OX6* and the WT; however, the degree of increase was less than that resulting from *PIN1a Ri2* (Figure 1u,w). These data suggest that *LPA1* positively controls resistance to SBD via initiation of *PIN1a*. In addition, the regulation of the tiller angle by *LPA1* may partially regulate resistance to SBD via PIN1a*‐*dependent auxin transport. We further examined the effect of auxin on resistance to SBD. 3‐Indole acetic acid (IAA), a natural form of auxin, was exogenously applied to plants, after which *R. solani* AG1‐1A was inoculated. The data showed that IAA treatment enhanced rice resistance to SBD (Figure 1x,y). Next, the endogenous IAA levels of the WT, *LPA1 OX5* and *LPA1 OX6* plants were measured. The data demonstrated that *LPA1* overexpressors contain higher levels of IAA than that of WT plant leaves (Figure 1z), implying that *LPA1* might activate *PIN1a* to accumulate more IAA.

Overall, our analyses identified that *LPA1* overexpression enhanced planting density by decreasing tiller and lamina joint angles. However, strong lines decreased tiller number and seed weight. The *LPA1* overexpression lines with moderate expressions not only sustained normal tiller angle, but also increased resistance to SBD, a major disease affecting rice cultivation. The molecular and biochemical data showed that *LPA1* triggers *PIN1a* via promoter binding. Interestingly, *PIN1a* controls tiller angle and resistance to SBD, and genetic combination between *LPA1* overexpressor and *PIN1a* knock‐down mutants revealed that mediation of planting density and resistance to SBD through overexpression of *LPA1* requires *PIN1a*. Exogenous auxin treatment enhanced rice resistance to SBD and resulted in *LPA1* overexpressors accumulating higher IAA than that of the WT. *Rhizoctonia solani* AG1‐1A‐mediated induction of Pathogen resistant genes *PBZ1* and *PR1b* levels were higher in *LPA1 OX* and *PIN1a OX* while lower in *PIN1a* RNAi lines than in wild‐type one, implying that *LPA1* might control auxin transport via initiation of *PIN1a* to increase planting density and activate plant defense gene expressions.
